# Does Thyroid Dysfunction Have a Role in the Etiology of Vocal Fold Edema?

**DOI:** 10.3390/clinpract15110214

**Published:** 2025-11-18

**Authors:** Alenka Kravos, Ksenija Gersak, Simona Gaberscek, Irena Hocevar-Boltezar

**Affiliations:** 1 University Division of Otorhinolaryngology, Head and Neck Surgery, University Medical Centre Maribor, Ljubljanska 5, 2000 Maribor, Slovenia; 2 Faculty of Medicine, University of Maribor, 2000 Maribor, Slovenia; 3 Department of Obstetrics and Gynecology, University Medical Centre Ljubljana, Zaloska 7, 1525 Ljubljana, Slovenia; ksenija.gersak@mf.uni-lj.si; 4 Faculty of Medicine, University of Ljubljana, 1000 Ljubljana, Slovenia; 5 Division of Nuclear Medicine, University Medical Centre Ljubljana, Zaloska 7, 1525 Ljubljana, Slovenia; simona.gaberscek@kclj.si; 6 Department of Otorhinolaryngology and Cervicofacial Surgery, University Medical Centre Ljubljana, Zaloska 2, 1525 Ljubljana, Slovenia

**Keywords:** vocal folds, Reinke’s edema, etiology, thyroid hormones, hypothyroidism, smoking

## Abstract

Background/Objectives: Previous studies have shown that hypothyroidism with myxedema may significantly affect the vocal folds. The aim of this study was to investigate thyroid dysfunction and other possible risk factors, including smoking, for the development of Reinke’s edema (RE) in a group of men. Methods. Serum levels of thyroid-stimulating hormone (TSH), free thyroxine (fT4), and free triiodothyronine (fT3) were measured in a group of men with first occurrence or recurrent RE and in age- and gender-matched control subjects without laryngeal pathology. All participants completed a questionnaire on other possible etiologic factors for the development of RE, especially smoking. Risk factors were compared between the two groups. Levels of TSH and thyroid hormones were also compared between patients with recurrent disease and those with a first occurrence of RE. Results. A total of 53 men with RE (mean age 53.82 years) and 45 controls (mean age 57.71 years) completed the study protocol. TSH and thyroid hormone levels were within the normal limits in all participants. Serum fT3 levels were significantly higher in patients with RE compared to controls (*p* = 0.034). After univariate analysis, all risk factors were included in a multivariate regression model. Only smoking remained a significant variable. Thyroid hormone levels did not differ between patients with first-onset RE and those with recurrent RE. Conclusions. An association between hypothyroidism and the development of RE was not confirmed. The significantly higher fT3 levels in patients compared to controls were still within the normal range and may reflect normal variation. Regression analysis of possible risk factors for RE showed the primacy of smoking.

## 1. Introduction

Reinke’s edema (RE) is a benign lesion of the subepithelial space of the vocal folds. It increases the mass of the vocal folds and is the main reason for the typical deep and hoarse voice characteristic of patients with RE [1]. In terms of risk factors, smoking is a known etiologic factor for RE [1]. Laryngopharyngeal reflux, overuse and misuse of the voice, and an unfavorable workplace microclimate are other possible factors [2].

The typical patient with RE is a middle-aged woman [3]. The gender difference in the incidence of RE can be attributed to the characteristic low voice, which may be more disturbing for women than for men. This could be the reason why women seek medical help more frequently than men. On the other hand, the gender difference in the incidence of RE may also be due to endogenous factors (e.g., sex, thyroid or growth hormones), since smoking, the main etiologic factor for RE, is common in both genders. The levels of sex hormones can also cause swelling of the vocal folds and thus affect voice quality. It is known that mild edema of the vocal folds can occur in women shortly before menstruation. Swelling of the vocal folds is often observed in women experiencing voice complaints that occur after menopause and its accompanying changes in sex hormone levels [4]. In a recent study on serum sex hormone levels, a difference in sex hormone levels was also found in men with RE. Specifically, male patients with RE were found to have significantly higher serum levels of progesterone and testosterone than men without pathologic changes in the vocal folds [5].

It is well known that patients with thyroid disease often complain of voice problems [6]. In addition to other clinical signs and symptoms of hypothyroidism, a significant proportion of patients with mild thyroid dysfunction have been found to have changes in their voices [7,8,9,10,11,12]. The onset of hoarseness is gradual and progressive, resulting in a deep, raspy voice that tires easily [3]. There have also been a few reports on laryngeal myxedema in patients with hypothyroidism [10]. On the other hand, some authors have also noted vocal pathology and changes in voice characteristics in female patients with elevated thyroid hormone levels [9].

Thyroid hormones are necessary for normal human development, growth, and the function of most organs. These hormones act on tissues by binding to their receptors, which are usually localized in the nuclei of target cells in various organs, producing genomic effects [13]. Thyroid hormone receptors (TRs) have been found in both female and male larynxes. TRs-alpha receptors are located in the fibrous connective tissue of the lamina propria, cartilage, and glandular elements, while TRs-beta receptors are only present in the fibrous connective tissue of the lamina propria. No receptors were found in the mucous membrane of the airways or in the laryngeal muscles [14].

The effect of thyroid hormones on laryngeal tissue is already known. The larynx is a target organ for thyroid hormones throughout the lifespan. Together with the sex hormones, the genomic effect of thyroid hormones dominates during the growth phase of the larynx [15]. Later, the non-genomic action of thyroid hormones also becomes important and is characterized by the rapid action of thyroid hormones via receptors on the endothelial cell membrane and in the cytosol [16].

The most prominent histologic change in RE is edema extending over the membranous portion of the vocal fold [1]. There are several theories about the causes of hoarseness in patients with hypothyroidism and swelling of the vocal folds. The deposition of proteoglycans is considered characteristic of myxedema in hypothyroidism [8]. Thus, the vocal folds may also become myxedematous and thickened [10]. Thyroid hormones, especially triiodothyronine (T3), can act on the endothelial cells of the vessels in Reinke’s space, which influences vascular tone and subsequently the permeability of the vessels [17]. Increased vascular permeability leads to fluid leaking from the vessels into the tissue, i.e., to edema.

Thyroid hormones have genomic and non-genomic effects on the endothelial cells in the vessels of the vocal folds. The genomic effect increases the transcription of nitric oxide (NO) synthase in the endothelial cell, which leads to increased production of the vasodilatory substance NO. The non-genomic effect is mediated by T3 membrane and cytosolic receptors, which cause a direct increase in NO and relaxation of the vascular smooth muscle cells. The end result of both is vasodilation [18].

Previous data showed the presence of voice problems, a lower voice quality with corresponding voice characteristics, and pathological changes in the vocal folds in patients with hypothyroidism or hyperthyroidism. However, the results of these studies were not always consistent regarding confirming the presence of dysphonia and vocal fold edema [7,8,9,10,11]. Although thyroid dysfunction has been associated with an impaired voice quality for many years in otorhinolaryngological clinical practice, few studies have been conducted in patients with Reinke’s edema of the vocal folds. Since fluctuations in female sex hormones can also affect the vocal folds, a study with male participants would appear to be the best way to investigate the role of thyroid hormones in the development of Reinke’s edema.

The aim of our study was to investigate thyroid function in male patients with known Reinke’s edema of the vocal folds, including patients with first occurrence and/or recurrent disease. In addition to thyroid function, other known risk factors were also investigated.

## 2. Materials and Methods

### 2.1. Participants

This non-randomized, prospective study was conducted over a period of 3 consecutive years (2011–2013). Consecutive male patients with RE attending the phoniatric outpatient clinic of the Department of Otorhinolaryngology and Head and Neck Surgery of the University Medical Centre Maribor were invited to participate in the study. Only patients who required microsurgical intervention on the vocal folds and whose histopathological examination of the vocal fold tissue sample confirmed the clinical diagnosis were included in the study. Subjects who had a known and treated thyroid disease, a history of previous treatment for benign or malignant head and neck pathology (except recurrent RE of the vocal folds), or who did not give blood samples for thyroid hormones serum analysis were excluded from the study.

The control group consisted of age-matched (+/− 5 years) men who visited the general ear, nose and throat outpatient clinic of the same institution during the same period. They suffered from dizziness, earache, chronic rhinitis or headaches, but denied any voice problems. Only those without voice problems and with normal laryngoscopic findings were included. The exclusion criteria for the control group were previous treatment for benign or malignant head and neck pathology, including treatment for any laryngeal pathology, known thyroid disease, and incomplete assessment of thyroid hormone serum levels. Having recovered from COVID-19 was not an exclusion criterion for either group in the study population.

### 2.2. Study Protocol

Patients with RE and the control group completed the same questionnaire on various possible etiologic factors for RE (smoking, vocal load at work and/or improper vocal habits, unfavorable workplace microclimate). The study population was asked about symptoms of laryngopharyngeal and gastroesophageal reflux (feeling of a lump in the throat, hoarseness in the morning, frequent clearing of the throat, irritating cough after eating; heartburn, acid regurgitation into the mouth). Irritants or uncomfortable temperatures were considered an unfavorable microclimate in the workplace. If the participants stated that they had considerable vocal load at work and were often shouting or whispering in their free time, this was assessed as vocal overload or inappropriate vocal habits (vocal overuse/misuse). Information about their profession and education level, any previous surgical interventions, and the histologically proven recurrence of RE was taken from the medical records. Televideostroboscopy was used to accurately visualize the anatomy and function of the larynx, to confirm the diagnosis of RE or normal larynx, and to assess the grade of RE [19]. Perceptual assessment of the voice of the patients (Grade Roughness–Breathiness Scale) was performed by a phoniatrician on a four-point scale (normal voice–slight voice disorder–moderate voice disorder–severe voice disorder). The patients assessed their own voice quality on a similar four-point scale.

Serum levels of thyroid-stimulating hormone (TSH), free thyroxine (fT4) and free triiodothyronine (fT3) were determined. Serum samples were taken between 8 a.m. and 10 a.m. to exclude the influence of diurnal fluctuations in thyroid hormone levels. TSH, fT4, and fT3 concentrations were determined using radioimmunoassay systems (electrochemiluminescence method “ECLIA” with immunoassay analyzer “COBAS EE 411”, Roche Diagnostic, Mannheim, Germany; reference values: THS = 0.27–4.2 mU/L, fT3 = 11.9–21.6 pmol/L, fT4 = 3.1–6.8 pmol/L; coefficient of variation was below 8.1%).

### 2.3. Statistical Analysis

The study group and control group were compared with regard to known etiological factors for RE (smoking, symptoms of gastroesophageal and laryngopharyngeal reflux, improper vocal habits/vocal load at work, unfavorable microclimate at work) and the levels of TSH, fT4 and fT3. The binary risk factors for the development of RE were analyzed by a χ2 test and a Fisher exact test, as well as being included in a logistic regression model. Non-significant variables were removed one by one, removing the largest *p*-value first, until all remaining variables in the model were significant. A correlation between smoking (smokers and ex-smokers), current smoking (only current smokers without ex-smokers) and the serum levels of TSH, fT4 and fT3 was tested.

The values of TSH and thyroid hormones were also compared between the subgroup of patients with recurrent RE and the patients with a first occurrence of RE.

The statistical package SPSS 24.0 (SPSS Inc., Chicago, IL, USA) was used to perform the analyses. In addition to the previously mentioned statistical methods, a two-tailed *t*-test, a non-parametric Mann–Whitney test, and Spearman’s correlation coefficient were used. A *p*-value < 0.05 was considered statistically significant.

### 2.4. Ethical Aspects

This study was carried out in accordance with the Code of Ethics of the World Medical Association (Declaration of Helsinki). The study protocol was approved by the National Medical Ethics Committee of the Republic of Slovenia (Protocol No. 101/01/10, dated 16 March 2010). All subjects in the study group and the control group confirmed their willingness to participate in the study by providing written informed consent.

## 3. Results

There were 53 patients with RE and 45 control subjects included in the study. The patients with RE were 53.82 ± 9.89 years old (range 34–76 years), while the subjects in the control group were 57.71 ± 11.48 years old (range 31–80 years). There was no significant difference between the groups in terms of age (*p* = 0.075). The patients and the control subjects had 28 different occupations. In the RE group, 20 (37.74%) patients had only finished elementary school, 26 (49.06%) had finished high school, and 7 (13.46%) had a college education. Among them, there were 5 (9.43%) subjects with occupations involving vocal load (sport trainer, salesman). In the control group, there were 16 (35.56%) subjects who had finished elementary school, 17 (37.78%) subjects with a high school diploma, 8 (17.78%) subjects with a college degree, and 4 (8.89%) subjects who did not share information about their occupation and level of education. Among them, there were 7 (15.56%) subjects who were professional voice users (manager, teacher, salesman). The groups did not significantly differ in educational level (*p* = 0.846) or in the number of subjects with vocal load at work (*p* = 0.357).

Among the 53 patients with RE, there were 8 (15.9%) patients with recurrent disease who had undergone RE surgery 3–15 years (mean 6.29 ± 1.88 years) before the current microsurgical treatment.

Televideostroboscopy of the larynx showed Grade 2 RE in 6 (11.32%) patients, Grade 3 in 39 (73.58%) patients, and Grade 4 in 8 (15.09%) patients. All control subjects had normal laryngoscopy findings.

All patients’ voices were abnormal. The grade of dysphonia was assessed as mild in 5 (9.43%) patients, moderate in 43 (81.13%) patients, and severe in 5 (9.43%) patients. Roughness was assessed as mild in 6 (11.32%) patients, moderate in 43 (81.13%) patients, and severe in 4 (7.54%) patients. Breathiness was assessed as absent in 42 (79.2%) patients, mild in 8 (15.09%) patients, and moderate in 3 (5.66%) patients.

The patients assessed their voices as mild dysphonia in 4 (7.54%) cases, moderate dysphonia in 42 (79.25%) cases, and severe dysphonia in 7 (13.21%) cases.

All participants from the control group had normal voices according to the phoniatrician’s and their own assessment.

When comparing various possible etiologic factors for the development of RE, only smoking was significantly more common in patients with RE than in control subjects (Table 1). In the RE group, there were 2 (3.77%) non-smokers, 31 (58.49%) smokers and 17 (32.08%) ex-smokers who had given up smoking at least 6 months previously. In the control group, there were 33 (73.33%) non-smokers, 5 (11.11%) smokers and 4 (8.89%) ex-smokers. Three patients (5.66%) with RE and three control (6.67%) subjects did not want to give information about their smoking habits.

There were no significant differences between the groups in terms of symptoms of laryngopharyngeal/gastroesophageal reflux. Among the men with RE, there were 34 (64.15%) subjects who had symptoms of gastric reflux above the lower esophageal sphincter, and 32 (71.11%) subjects in the control group (Table 1).

Vocal load at work or during leisure time was present in four (7.54%) men with RE (in two of them due to noise at work) and in one (2.22%) control subject. The habit of frequently speaking loudly or shouting was characteristic of the same four (7.54%) men with RE and an additional three (6.67%) control subjects. Overuse or misuse of the voice due to occupation and/or inappropriate vocal habits were grouped into one category (vocal overuse/misuse) (Table 1).

Workplace irritants were reported by 31 (58.49%) men with RE and 25 (5.56%) controls. Seven (13.21%) men with RE and seven (15.56%) controls worked in a room with a too high or too low temperature. Both categories were combined into one category (unfavorable microclimate) for statistical analysis (Table 1).

When we entered risk factors for the development of RE (smoking, symptoms of gastroesophageal/laryngopharyngeal reflux, vocal overuse/misuse, unfavorable microclimate) into a multiple logistic regression model, only smoking emerged as a significant variable. Non-significant variables were removed one by one, first symptoms of reflux (*p* = 0.398), then unfavorable microclimate (*p* = 0.109), and finally vocal overuse/misuse (*p* = 0.068). Only smoking remained significant throughout the process of analysis, with B = −4.477, *p* = 0.000, and a 95% confidence interval from 0.002 to 0.056 at the end.

The serum levels of TSH and free thyroid hormones in the patients with RE and in the control subjects are shown in Table 2. In both groups, the serum levels of TSH, fT4 and fT3 were within the normal reference range. The fT3 level was significantly higher in the group of patients with RE than in the control group, while the TSH and fT4 levels did not differ between the two groups.

In order to verify a possible correlation between smoking and the serum levels of TSH and thyroid hormones, Spearman coefficients were calculated. No significant correlations were found between smoking and levels of TSH (rho −0.018, *p* = 0.865), fT4 (rho −0.081, *p* = 0.557) and fT3 (rho 0.229, *p* = 0.090). There were also no significant correlations between current smoking and levels of TSH (rho −0.086, *p* = 0.422), fT4 (rho 0.011, *p* = 0.936) and fT3 (rho 0.222, *p* = 0.100).

There were eight patients with recurrent disease. They had undergone laryngeal surgery for RE 3–15 years before the current microsurgical treatment. Smoking habits in those with recurrent disease did not significantly differ from patients with first occurrence of the disease. All eight patients were smokers, and of these seven patients were current smokers (*p* = 0.156).

The serum levels of TSH, fT4 and fT3 did not differ significantly between the patients with recurrent RE and the patients with first occurrence of the disease (Table 3).

## 4. Discussion

In the present study, we investigated the role of thyroid function in the etiology of RE in men by determining serum levels of TSH, fT4, and fT3. We decided to include only men, although RE is much more common in female patients. In women, thyroid dysfunction may also be present unrelated to laryngeal pathology. The effect of sex hormones on the vocal folds in women is well known and has been documented. This effect is present throughout the menstrual cycle and also varies during perimenopause and early post-menopause, depending in particular on BMI, alcohol consumption, and smoking habits [20]. For these reasons, we only included men in the study.

We found that serum fT3 levels were significantly higher in men with RE than in those without laryngeal pathology, suggesting that fT3 may play a role in the development of RE. However, our results did not confirm an association between thyroid dysfunction and RE, as all patients with RE had normal serum levels of TSH, fT4 and fT3. We hypothesized that patients with RE would have higher TSH levels and lower thyroid hormone levels than controls. This hypothesis is based on several reports of voice disorders in patients with just mild thyroid dysfunction [7,8,9,10]. Canaris et al. identified a hoarse voice as a symptom strongly suggestive of hypothyroidism. A hoarse voice was found in 17% of patients with hypothyroidism and only 4% of control subjects [21]. Ingbar and Woeber described a change in voice quality in 52% of patients with hypothyroidism [22]. Contrary to these studies, our results confirm the findings of White’s study, which found no clear association between hypothyroidism and RE [11]. It is possible that the higher levels of fT3 in the study groups are merely a consequence of variations within normal limits.

The most striking histological feature of RE is the accumulation of edema in Reinke’s space. The main components of the extracellular matrix of Reinke’s space are hyaluronic acid (HA) and fluid [23,24]. In the case of hypothyroidism, myxedema of the laryngeal mucosa could cause the accumulation of both components in the Reinke’s space of the vocal folds. Our results do not show any of the hormone changes characteristic for hypothyroidism, but they do confirm the known fact that smoking is the most important etiologic factor for RE. Indeed, the effects of smoking can also contribute to the accumulation of HA and fluid in Reinke’s space. Smoking represents a chemical stress that leads to greater amounts of NADPH and the induction of HA synthases 2 gene expression in vascular smooth cells [25]. Almost all our RE patients were smokers (or ex-smokers) and were thus exposed to chemical stress for a considerable period in their lives. Therefore, it is possible that the elevated NADPH level increased the production of HA, which contributed significantly to the development of Reinke’s edema of the vocal folds. Additional, more complex analyses at the molecular level, including the determination of NADP levels in the tissue, would be necessary for a direct proof of our hypothesis.

The other important component of increased extracellular matrix in the Reinke’s space in RE is fluid. In RE, the vessels in Reinke’s space are dilated and have a thickened wall. Endothelial disruptions are also present in RE, but are very limited and also very rare [1,23]. Earlier theories about the development of RE assumed that these vessel wall disruptions were the only spaces through which fluid could leak and were thought to be the result of mechanical stress from excessive vocal use [23]. In our study, excessive voice use or misuse was reported by only four patients with RE and four controls. Therefore, vocal overuse/misuse seems not to be a crucial factor in the development of RE.

However, more recent studies have reported an upregulation of water channels in fibroblasts in Reinke’s space leading to edema formation [26]. During vasodilatation, several protein structures in the intercellular spaces are activated. Fluid leakage from the vessels is selective and is upregulated by structures in the intercellular spaces (tight junctions) [17,25]. One of strongest vasodilators is fT3, which could play an important role in fluid leakage from the vessels [16]. Although fT3 levels were significantly higher in patients with RE than in control subjects, they were still within the normal range. Therefore, fT3 may not be an important factor causing fluid leakage into the Reinke’s space of the vocal fold leading to RE.

Smoking, however, does contribute to the development of RE in several ways. It causes oxidative stress, which not only leads to increased HA production but also to endothelial dysfunction, which manifests itself in a narrowing of the vascular wall cells. The intercellular spaces then enlarge and allow fluid to leak out of the vessels into the Reinke’s space [27].

It has been reported that smoking can affect fT3 levels, as nicotine activates the sympathetic nervous system and stimulates T3 synthesis [28]. On the other hand, some other components of tobacco smoke impair the synthesis of thyroid hormones, and thus smoking is not thought to have a significant effect on free thyroid hormone levels [29]. In short, authors disagree on the effects of smoking on thyroid hormone levels [30]. In our study, fT3 levels were significantly higher in patients with RE (90.6% smokers, 58.5% current smokers) compared to controls (20% smokers, 11.1% current smokers). According to the results of previous studies, smoking is the main risk factor for RE and likely also for the recurrence of the disease [2,24]. A correlation analysis was performed in order to test the possible impact of smoking or current smoking on the levels of thyroid hormones. No significant correlation was found, thus confirming that smoking has no influence on thyroid hormone production.

We found similar levels of TSH and free thyroid hormones in patients with RE recurrence as in patients with a first occurrence of RE. They were all smokers, with seven being current smokers. We can therefore conclude that the level of thyroid hormones is not important for the recurrence of RE. Smoking is a characteristic of all these patients and is an important factor in the development of the first occurrence and the recurrence of RE.

## 5. Conclusions

Serum levels of fT3 were significantly higher in men with RE than in control subjects without laryngeal pathology, although they were still within the normal range. Thyroid hormones can have an influence on the vessels in Reinke’s space, and therefore, theoretically, vasodilatation regulated by fT3 is possible. However, the results of our study could not confirm an association between hypothyroidism and RE. As for the higher fT3 levels in patients with RE, it is possible that this result is a consequence of a still-normal variation in thyroid hormone production. According to our results, smoking was confirmed as the only significant risk factor for the development of RE with no significant influence of thyroid hormone production.

## Figures and Tables

**Table 1:** Risk factors for Reinke’s edema of the vocal folds in male patients with Reinke’s edema (RE) (N = 53) and in male controls (N = 45).

Etiological Factor	RE	Control	*p*
Smoking	48 (90.57%)	9 (20%)	0.000
Current smoking	31 (58.49%)	5 (11.11%)	0.000
Symptoms of gastroesophageal/laryngopharyngeal reflux	34 (64.15%)	32 (71.11%)	0.385
Vocal overuse/misuse	4 (7.55%)	4 (8.89%)	0.898
Unfavorable microclimate	31 (58.49%)	27 (60%)	0.821

**Table 2:** Serum levels of thyroid-stimulating hormone (TSH) and thyroid hormones (free thyroxine—fT4, free triiodothyronine—fT3) in male patients with Reinke’s edema (RE) of the vocal folds (N = 53) and male control subjects without laryngeal pathology (control) (N = 45).

Hormone	Subjects	Mean	Standard Deviation	*p*
TSH	RE	1.71 mU/L	0.79 mU/L	0.819
	Control	1.56 mIU/L	0.78 mIU/L	
fT4	RE	16.02 pmol/L	2.72 pmol/L	0.270
	Control	15.97 pmol/L	4.21 pmol/L	
fT3	RE	5.29 pmol/L	0.51 pmol/L	0.034
	Control	4.89 pmol/L	0.82 pmol/L	

**Table 3:** Serum levels of thyroid-stimulating hormone (TSH) and thyroid hormones (fT4, fT3) in men with recurrent Reinke’s edema (recurrent RE) (N = 8) and in those with first onset of the disease (first RE) (N = 45).

Hormone	Subjects	Mean	Standard Deviation	*p*
TSH	Recurring RE	1.92 mU/L	0.89 mU/L	0.230
	First occurrence of RE	1.69 mU/L	0.67 mU/L	
fT4	Recurring RE	16.84 pmol/L	1.56 pmol/L	0.395
	First occurrence of RE	15.87 pmol/L	3.11 pmol/L	
fT3	Recurring RE	5.32 pmol/L	0.38 pmol/L	0.784
	First occurrence of RE	5.26 pmol/L	0.57 pmol/L	

## Data Availability

The datasets presented in this article are not readily available due to technical limitations. Requests to access the datasets should be directed to the corresponding author.

## Abbreviations

The following abbreviations are used in this manuscript:

RE	Reinke’s edema
TSH	Thyroid-stimulating hormone
fT4	Free thyroxine
fT3	Free triiodothyronine
TRs	Thyroid hormone receptors
NO	Nitric oxide
T3	Triiodothyronine
HA	Hyaluronic acid
NADPH	Nicotinamide adenine dinucleotide phosphate
